# The relationship between anxiety and parent-child attachment and psychological resilience among Chinese college students: take 100 thousand college students as sample

**DOI:** 10.3389/fpsyt.2025.1667736

**Published:** 2026-01-07

**Authors:** Zhe Li, Yu-Yu Zhao, Xin Li, Ke Liu, Dong-Hua Tian, Su-Xia Li, Xiang-Yang Zhang

**Affiliations:** 1 School of Government, Beijing Normal University, Beijing, China; 2 National Institute on Drug Dependence and Beijing Key laboratory of Drug Dependence Research, Peking University, Beijing, China; 3 Department of Neurobiology, Peking University Health Science Center, Beijing, China; 4 School of Chemistry and Biological Engineering, University of Science and Technology, Beijing, Beijing, China; 5 School of Sociology, Beijing Normal University, Beijing, China; 6 Hefei Fourth People's Hospital, Anhui Mental Health Center, Affiliated Psychological Hospital of Anhui Medical University, Hefei, China

**Keywords:** anxiety, depression, parent-child attachment, psychological resilience, college student

## Abstract

**Background:**

Previous studies on the relationship between good parent-child attachment, psychological resilience with mental health, have limitations such as small sample sizes and specific regions or universities, which lack universality. This study aims to survey a total of 100,253 college students across China to clarify the correlation between parent-child attachment, psychological resilience and mental health among college students.

**Methods:**

Online questionnaires were performed from April 13 to 23, 2020 in China, used 10-item Depression Self-Rating Scale, Generalized Anxiety Disorder Scale (GAD-7), Resilience Scale-Simplified (CD-RISC-10), and Parental Attachment Items in the Parent-Peer Attachment Scale (IPPA).

**Results:**

In total, 100,253 university students completed this survey. The detection rate of anxiety symptoms and depression symptoms were 42.74% and 44.25%, respectively. The parent-child attachment and psychological resilience among them were in a medium level. Anxiety symptoms, rather than depression symptoms were closely related to the quality of parent-child attachment and psychological resilience. However, there was no correlation between depression scores and parent-child attachment, as well as psychological resilience.

**Conclusion:**

Our findings indicate that both depression and anxiety symptoms in college students’ population are high, and suggest that college students may have different etiology and pathogenesis of depression and anxiety. The treatment strategies for these two different types of symptoms for college students’ population, especially the psychological treatment strategies, should be differentiated accordingly.

## 1 Background

Depression and anxiety disorders are two of the most prevalent mental health disorders globally, with significant impacts on an individual’s health and social functioning (1). According to the Chinese Centre for Disease Control and Prevention (CDC) (2) and the National Blue Book on Depression 2022 (3), the number of people suffering from depression in China is as high as 95 million, accounting for about one-third of the global population of depressed individuals. A survey of China’s mental health showed that the prevalence of anxiety disorders is the highest among all major mental disorders in China, even surpassing depression, with a lifetime prevalence of about 7.6% and an annual prevalence of about 4.98% among Chinese adults (4), and approximately 40 million Chinese people suffering from anxiety disorders each year. Clinical experience and empirical data suggest that depression and anxiety symptoms are highly co-morbid with the median of the pooled estimates (OR) was 6.1 (range 1.5-18.7) (5), severely affecting people’s social functioning and causing economic burdens (6).

Chinese college students face challenges such as academic pressure, interpersonal relationships, and career choices, as well as a growing sense of independence, sometimes they face mood swings, coupled with a lack of cognitive level and experience, which may make them more susceptible to depression and anxiety, negatively affecting their academic performance, interpersonal relationships, physical health, sleep quality, and even increase the risk of suicide (7). A meta-analysis showed that the incidence of depression among Chinese college students has been increasing year by year, with prevalence rates ranging from 15% to 40%, which is higher than the general population of 5%~6% (8). Another meta-analysis indicated the overall prevalence of depression among Chinese college students in the last ten years of 31.38% (9). A prospective follow-up study conducted by Huang Yueqin’s team at Peking University showed that the cumulative prevalence of depressive symptoms, assessed by the Centre for Epidemiological Study-Depression Scale (CES-D), among undergraduate freshmen (including 391 males with an average age of 18.4 ± 0.90 years and 367 females with an average age of 18.1 ± 0.78 years) within two years after enrolment was 42% (10). The COVID-19 pandemic emerged at the end of 2019, prompting many countries to implement measures such as lockdowns, home quarantines, self-isolation, and social distancing (11), which resulted in significant public health challenges (12, 13). A survey of 12,922 undergraduates at a university in eastern China in 2022 showed that the prevalence of anxiety and depression among university students was 22.7% and 46.8%, respectively (14). Meanwhile, a meta-analysis showed that the pooled detection rate of anxiety symptoms among Chinese university students was 21.51% (15). In general, the incidence of depression and anxiety among Chinese college students is relatively high, and it has shown an upward trend in recent years. Therefore, universities and society need to strengthen their attention and support for the mental health of college students to cope with this increasingly serious problem. For instance, establishing monitoring mechanisms in university campuses and providing convenient channels for psychological counseling and therapy, etc.

The concept of attachment was first proposed by Bowlby (16). With the development of the time, the definition of attachment has also been constantly updated. This study refers to Ju Xiaoyan’s definition of attachment: “Attachment refers to the deep, stable, and continuously developing emotional connection formed with a person who has played an important role in the development of his or her life” (17). Parent-child attachment refers to the deep-seated emotional relationship that is established by the continuous interaction between the child and the parents in their daily lives, and this connection is not easily changed (18). Studies have shown that the anxiety level of college students is closely related to the quality of their parent-child attachment. For example, those undergraduate students (Mean age = 19.27, SD = 0.97) with higher quality of parent-child attachment tend to be better able to cope with academic and social stresses and exhibit lower levels of anxiety (19). Parental marital conflict decreases the quality of parent-child attachment and increases anxiety in college students, while high-quality attachment helps reduce social anxiety symptoms (20). In addition, the quality of parent-child attachment has a significant predictive effect on the emotional stability of college students (21). High-quality attachment contributes to the emotional stability of college students, while poor emotional stability are more likely to develop their anxiety (22). College students with secure attachment typically exhibit higher levels of self-identity and lower levels of anxiety (23). While, college students with insecure attachment may face more social anxiety and adjustment problems (24). It is reported that the inconsistent paternal and maternal attachments were not significantly associated with depressive symptoms in sons, whereas insecure maternal attachment was more closely related to daughters’ depressive symptoms (25). The attachment to father emerged as significant protective factors of anxiety and the attachment to father and/or mother was significantly associated with decreased depressive symptoms (26). Based on these pieces of evidence, a good attachment relationship is helpful for emotional regulation.

Resilience refers to the individual’s ability to bounce back in the face of setbacks (27). Studies have found that resilience plays an important mediating role in the relationship between parent-child attachment and college students’ mental health. A good parent-child attachment relationship can reduce college students’ anxiety by improving resilience (28). At the same time, the psychological resilience model and the social construct model found that “acceptance and positive attachment relationships promote the construction of resilience” (29, 30), indicating that good attachment is an important protective factor for the development of individual resilience. Previous studies also suggested that resilience played a moderating role, that is students with higher psychological resilience manifested a significantly lower correlation between stress and depression, highlighting its protective function (31, 32). A previous longitudinal study (30) found that children with high levels of resilience had more ecosystem factors that contribute to physical and mental development, such as better quality care, higher sense of self-worth, and more support from family and friends, so resilience has a positive predictive effect on mental health (33). A cross-sectional study involved undergraduate medical colleges students revealed that resilience was negatively correlated with both stress and depression (34). Previous studies have also revealed that adolescent resilience has a significant impact on academic achievement (35), and there is a significant negative correlation between resilience and students’ learning burnout (36). At the same time, the quality of resilience affects individuals to mobilize their own resources to cope with stress and adapt to change, and individuals with high levels of resilience are more likely to maintain good mental health when coping with stress (37).

Although studies have explored the relationship between parent-child attachment, resilience, depression and anxiety, current research still has some limitations. For example, most of the existing research samples are selected from specific regions, populations or universities, and the research methods are mostly meta-analyses or cross-sectional surveys with small sample sizes, which lack universality. Therefore, this study surveyed a sample of 100,253 college students in China, first to clarify the incidence of depression and anxiety in college students. Second, to clarify the correlation between parent-child attachment, resilience, anxiety and depression among college students.

## 2 Materials and methods

### 2.1 Participants

All the participants were from 23 provinces, 4 municipalities directly under the Central Government, 5 autonomous regions and 1 special administrative region across the China. They covered all types of universities and colleges in China, totaling 816 higher education institutions in China, including 460 colleges and 356 universities.

### 2.2 Measurement

#### 2.2.1 Demographics

We collected demographic characteristics such as age, school and region from all participants.

#### 2.2.2 The 10-item of center for epidemiological survey, Depression scale (CES-D-10)

Depression was assessed using the Chinese version of CES-D-10 (38). The scale was developed by Andresen and his colleagues (39). There is a total of 10 items, such as ‘I’m troubled by some trivial matters’, using a Likert 4-point score ranging from 0 [little or nothing (<1 days)] to 3 [most of the time (5–7 days)] to assess the frequency of symptoms in the past two weeks. CES-D-10 includes 2 items on positive impact, which are scored backwards. A score of 10 or more on CES-D-10 was considered to have depressive symptoms (39). The scale has a Cronbach’s alpha value of 0.752 in this study.

#### 2.2.3 The 7-item of generalized anxiety disorder scale (GAD-7)

Anxiety was assessed using the Chinese version of GAD-7 (40). The scale has a total of 7 items, such as ‘Feeling nervous, anxious or eager’, with a Likert 4-point score ranging from 0 (not at all) to 3 (almost daily) and is used to assess how often patients have experienced anxiety symptoms over the past two weeks. A score of 5 or over on GAD-7 was considered to have anxious symptoms (41). The scale has a Cronbach’s alpha value of 0.920 in the present research.

#### 2.2.4 The 10-item of connor-davidson resilience scale (CD-RISC-10)

The CD-RISC-10 was simplified by Campbell-sills et al. on a 25-item of CD-RISC (42), the Chinese version was revised by Zhang Danmei et al. (43). This scale consisting of 10 items, such as ‘When changes occur, I can adapt’, which is scored on a Likert 5 scale, ranging from 0 (never) to 4 (always), and is used to assess the subjects’ psychological resilience. The total score is added by each of the item, the higher the total score, the stronger the psychological resilience. The scale has a Cronbach’s alpha value of 0.910 in this study.

Those who scored 15% or less of and scored 85% or higher of the total scores on psychological resilience (total score of 40 points) respectively, was set as a low score or a high score.

#### 2.2.5 The inventory of parent and peer attachment

The IPPA scale was developed by Armsden et al. (44), the Chinese version was revised by Wang Shuqing (45). It including three subscales of maternal attachment, paternal attachment and peer attachment. The scale is scored on a 5-point scale, from “very consistent” to “strongly non-compliant”, divided into three dimensions: trust (for example, ‘Father/mother accepts me as I am now’), communication (for example, ‘I told my father/mother about the problems and difficulties I was facing’), and estrangement (for example, ‘Discussing my problems with my father/mother made me feel ashamed or foolish’) (reverse scoring). The total score is added by each of the item, with higher total scores representing higher attachment levels. The Cronbach’s alpha value of the maternal attachment and paternal attachment sub-scales was 0. 87, 0. 89. In this study, we just used the maternal attachment and paternal attachment sub-scales.

Those who scored 15% or less of and scored 85% or higher of the total scores on parent-child attachment (total score of 50 points) respectively, was set as a low score or a high score.

### 2.3 Procedure

The survey was conducted from April 13 to 23, 2020, in collaboration with the Chinese Psychological Association. The Chinese Psychological Association is an academic society affiliated with the Institute of Psychology, Chinese Academy of Sciences. Under the Psychological Crisis Intervention Committee of the Chinese Psychological Association, there is a National University Psychology Committee Collaborative Group, including 177 colleges and universities in China. Each class in each university was set up 1–2 psychological monitors responsible for contacting the whole class and concentrating the mental health states of students. Every university has aggregate hundreds or even thousands psychological monitors, who are led and trained by the National University Psychology Committee Collaborative Group. This questionnaire survey was sent by the National University Psychology Committee Collaborative Group to the psychological monitors, and then introduced to the students by the psychological monitors. All participants signed informed consent forms to participate in the survey after the beginning of school, and participation was voluntarily. College students who volunteered to participate in the survey could click a link to enter the online questionnaire system. The participants were notified that online and in-person psychological counselling services were available if needed after the survey.

All authors assert that all procedures contributing to this work comply with the ethical standards of the relevant national and institutional committees on human experimentation and with the Helsinki Declaration of 1975, as revised in 2013. All procedures involving human subjects were approved by the ethics review committee of the Institute of Psychology, Chinese Academy of Sciences and approval number was H19068.

### 2.4 Statistical analysis

The SPSS 26 software was used for statistical analysis. Continuous variables are presented as the means ± standard deviations (SDs) and categorical variables are presented as percentages with 95% confidence intervals (CIs). The Pearson correlation method was applied to assess the relationships among various variables. The correlation *P* values was significant at the 0.01 level (two-tailed).

## 3 Results

### 3.1 Descriptive statistics

A total of 100,257 college students participated in the online survey, 4 participants were excluded due to incomplete scale data, and the final number of participants was 100,253. **Table 1** showed that the mean age of participants was 20.09 ± 1.37, ranging from 16 to 27 years. The mean score on CES-D-10 was 7.56 ± 5.27, with the number of subjects with depressive symptoms was 44359, and the detection rate was 44.25% (95% CI [41.11%, 47.39%]). The mean score on GAD-7 was 2.50 ± 3.56, with the number of subjects with anxiety symptoms was 42853, and the detection rate was 42.74% (95% CI [40.58%, 44.90%]). The mean score on the Parent-Child Attachment was 29.94 ± 4.41, and on the psychological resilience was 26.68 ± 7.32. According to the predefined criteria the average quality of parent-child attachment and psychological resilience of college students was in the medium level.

**Table 1:** Demographic and clinical characteristics of participants.

Characteristics	Mean ± SD	Number of Cases	Incidence Rate (%, [95%CI])
Age	20.90 ± 1.37		
CES-D-10	7.56 ± 5.27	44359.00	44.25% [41.11%, 47.39%]
GAD-7	2.50 ± 3.56	42853.00	42.74% [40.58%, 44.90%]
CD-RISC-10	26.68 ± 7.32		
IPPA	29.94 ± 4.41		

CES-D-10, 10-item Center for Epidemiological Studies Depression Scale; GAD-7, Generalized Anxiety Disorder Scale; CD-RISC-10, 10- item Connor- Davidson resilience scale; IPPA, Inventory of Parent and Peer Attachment; SD, Standard deviation.

### 3.2 Correlation of scores on four scales among all participants

**Table 2** showed that among all participants, scores on GAD-7 were negatively correlated with scores on parent-child attachment (r = -0.048, *p* < 0.001) and on psychological resilience (r = -0.284, *p* < 0.001), respectively. The scores on parent-child attachment was positively correlated with scores on psychological resilience (r = 0.274, *p* < 0.001).

**Table 2:** Correlation between total GAD-7 scores with IPPA and CD-RISC-10 scores.

		GAD-7	IPPA	CD-RISC-10
GAD-7	*r*	1	-.048**	-.284**
	*p*		.000	.000
IPPA	*r*	-.048**	1	.274**
	*p*	.000		.000
CD-RISC-10	*r*	-.284**	.274**	1
	*p*	.000	.000	

**At the 0.01 scale (two-tailed), the correlation is significant. GAD-7, Generalized Anxiety Disorder Scale; IPPA, Inventory of Parent and Peer Attachment; CD-RISC-10, 10- item Connor- Davidson resilience scale.

Contrary to our expectations, **Table 3** showed that scores on CES-D-10 did not correlate with scores on parent-child attachment (r = 0.004, *p* = 0.17), nor did with scores on psychological resilience (r = 0.004, *p* = 0.203). The scores on parent-child attachment was still positively correlated with scores on psychological resilience (r = 0.274, *p* < 0.001).

**Table 3:** Correlation between total CES-D-10 scores with IPPA and CD-RISC-10 scores.

		CES-D-10	IPPA	CD-RISC-10
CES-D-10	*r*	1	.004	.004
	*p*		.170	.203
IPPA	*r*	.004	1	.274**
	*p*	.170		.000
CD-RISC-10	*r*	.004	.274**	1
	*p*	.203	.000	

**At the 0.01 scale (two-tailed), the correlation is significant. CES-D-10, 10-item Center for Epidemiological Studies Depression Scale; IPPA, Inventory of Parent and Peer Attachment; CD-RISC-10, 10- item Connor- Davidson resilience scale.

### 3.3 Correlation between anxiety scores, parent-child attachment and psychological resilience scores in anxious populations

In the population with anxiety symptoms, we further analyzed the correlation between scores on GAD-7 and scores on parent-child attachment and psychological resilience, respectively. **Table 4** indicated that scores on GAD-7 were negatively correlated with scores on parent-child attachment (r = -0.024, *p* < 0.001) and on psychological resilience (r = -0.218, *p* < 0.001), respectively. Scores on the parent-child attachment were also positively correlated with scores on the psychological resilience (r = 0.231, *p* < 0.001). This is consistent with the correlation found in the overall population.

**Table 4:** Correlation of GAD-7 scores, IPPA scores, and CD-RISC-10 scores in participants with anxiety symptoms.

		GAD-7	IPPA	CD-RISC-10
GAD-7	*r*	1	-.024**	-.218**
	*p*		.000	.000
IPPA	*r*	-.024*	1	.231**
	*p*	.000		.000
CD-RISC-10	*r*	-.218**	.231**	1
	*p*	.000	.000	

**At the 0.01 scale (two-tailed), the correlation is significant. GAD-7, Generalized Anxiety Disorder Scale; CD-RISC-10, 10- item Connor-Davidson resilience scale; IPPA, Inventory of Parent and Peer Attachment.

### 3.4 Correlation between anxiety scores and subscales of parent-child attachment in anxious populations

We further explored the correlation between scores on GAD-7 and scores on subscales of parent-child attachment in people with anxiety symptoms. **Table 5** showed that scores on GAD-7 negatively correlated with scores on communication subscale (r = -0.149, *p* < 0.001) and scores on trust subscale (r = -0.187, *p* < 0.001), respectively. While scores on GAD-7 positively correlated with scores on alienation subscale (r = 0.242, *p* < 0.001). Among the three dimensions of parent-child attachment, scores on trust subscale and communication subscale were positively correlated (r = 0.624, *p* < 0.01). Scores on alienation subscale were negatively correlated with scores on trust subscale (r = -0.442, *p* < 0.01) and on communication subscale (r = -0.384, *p* < 0.01), respectively.

**Table 5:** Correlations between GAD-7 scores and three dimensions of IPPA in participants with anxiety symptoms.

		GAD-7	Communication	Trust	Alienation
GAD-7	*r*	1	-.149**	-.187**	.242**
	*p*		.000	.000	.000
Communication	*r*	-.149**	1	.624*	-.384**
	*p*	.000		.000	.000
Trust	*r*	-.187**	.624**	1	-.442**
	*p*	.000	.000		.000
Alienation	*r*	.242**	-.384**	-.442**	1
	*p*	.000	.000	.000	

**At the 0.01 scale (two-tailed), the correlation is significant. GAD-7, Generalized Anxiety Disorder Scale.

## 4 Discussion

In this study, the detection rate of both anxiety and depression symptoms among Chinese college students is high, with a slightly higher detection rate of depressive symptoms (44.25%) than that of anxiety symptoms (42.74%). The severity of anxiety symptoms is closely related to the quality of parent-child attachment and psychological resilience. This indicates good parent-child attachment and psychological resilience can help reduce anxiety symptoms in college students, at the same time, good parent-child attachment may help improve the psychological resilience of them. In the three dimensions of parent-child attachment, communication and trust are positively correlated, while the both are negatively correlated with alienation, suggesting that factors such as warm family atmosphere, parental support and attention, encouragement and affirmation of children, and family stability can effectively reduce college students’ anxiety in the face of frustration and lower the likelihood of psychological problems. However, the severity of depression symptoms is not correlated to the quality of parent-child attachment and psychological resilience.

The detection rate of depressive symptoms and anxiety symptoms in our study were higher than previous studies (9, 46–48). This may be due to measurement tools varied between studies, our study using broad ‘symptom’ criteria (CES-D-10 and GAD-7), whereas some studies only counted ‘disorders’ that met a clinical diagnosis (46), resulting in large differences in detection rates. In this study, we used CES-D-10 score ≥ 10 as the cutoff point as the probability of depressive symptoms, GAD-7 score ≥ 5 as the cutoff point as the probability of anxiety condition. In addition, our data collection took place in the early stage of the COVID-19 pandemic, and the impact of COVID-19 on college students’ mental health can not ignore. The incidence of depression and anxiety symptoms in our survey is comparable to the reported incidence during the corresponding period. It is reported that the prevalence rates of depression and anxiety ranged from 9.0% to 65.2%, 6.88%-41.1%, respectively, in the early stage of COVID-19 pandemic; and 8.7% to 50.2%, 4.2%-34.6%, respectively, in the normalization stage of COVID-19 pandemic among Chinese college students (49). Accordingly, the detection rates of our sample are reasonable and credible.

This study found that the quality of parent-child attachment and psychological resilience of college students was in a medium level. The anxiety scores were negatively correlated with the scores of parent-child attachment and psychological resilience, respectively. There was a positive correlation between parent-child attachment and psychological resilience scores. Our results were consistent with previous studies (50), in which the parent-child attachment scores were negatively correlated with social anxiety scores, and positively correlated with psychological resilience scores among college students. Meanwhile, the psychological resilience mediates the relationship between parent-child attachment and social anxiety. It has been pointed out that mutual attachment between children and parents not only provides children with a sense of security and belonging, but also provides children with emotional support, social interaction and interpersonal opportunities (51). A secure and high-quality parent-child attachment relationship helps to reduce the likelihood of risky behaviors among adolescents (52–54), as well as helps to improve the individual’s ability to adapt to society, helping them to show a more positive and optimistic attitude in the social process (55). The higher the quality of parent-child attachment, the more advantageous individuals are in establishing new interpersonal relationships, and their satisfaction in the interpersonal process increases accordingly, therefore, they evaluate themselves and others more positively, and feel more confident and secure in the face of challenges and difficulties (56). When an individual is emotionally supported by a stable and high-quality attachment relationship, it will contribute to their overall relatively positive development. This kind of emotional support helps individuals to reduce anxiety in the process of exploring the outside world and to show higher self-confidence in the face of difficulties and challenges (56). Therefore, a good parent-child attachment relationship can effectively reduce the anxiety of college students in the face of setbacks and reduce the likelihood of psychological problems.

It is well known that the psychological resilience has a positive predictive effect on mental health (33). Individuals with high levels of psychological resilience have more ecosystem factors that contribute to physical and mental development, such as better quality of care, higher sense of self-worth, and more support from family and friends (33), they are more likely to maintain good mental health when coping with stress (37). Previous studies had also consistently found that individuals with high psychological resilience have higher levels of positive emotions, which facilitates effective coping with stress or adversity (57). The findings of the psychological resilience model and the social construct model (29, 30) indicated that individuals can effectively promote the construction of psychological resilience by establishing a positive attachment relationship in an accepting environment. This clearly reveals that good attachment relationships are a crucial protective factor in the development of psychological resilience. Previous study (58) also found a significant correlation between parental conflict, parent-child relationship and college students’ psychological resilience, suggesting that high-quality parent-child relationships can enhance psychological resilience. In view of this, our findings and previous findings all prove that psychological resilience and parent-child attachment jointly promote individuals’ mental health, and a good attachment relationship is an important protective factor for the development of individual psychological resilience. Factors such as a warm family atmosphere, parental support and attention, and family stability may help promote psychological resilience among college students to cope with stress and negative emotions.

This study also found that the three dimensions of parent-child attachment (communication, trust, and alienation) were significantly correlated with anxiety among Chinese college students. Anxiety scores negatively correlated with communication and trust scores, while, positively correlated with alienation scores. Between the three dimensions, trust scores were positively correlated with communication scores, while, negatively correlated with alienation scores. Meanwhile, communication scores were negatively correlated with alienation scores. Our results were consistent with the previous study (56), in which high levels of communication and trust were often associated with lower levels of anxiety, while high levels of alienation were associated with higher levels of anxiety. Parent-child communication and trust have a positive impact on college students’ adversity growth by enhancing self-identity, whereas parental alienation decreases self-identity and negatively affects college students’ adversity growth (59). Positive communication and mutual trust between parents and children help children to better adapt to their environment, achieve better grades, and have a higher sense of well-being (60, 61). A secure attachment relationship will have a positive impact on the child’s interpersonal communication, helping individuals to make correct judgments to others and respond in a timely manner (60, 62). In contrast, insecure attachment is associated with internalization problems, anxiety or depression, and the onset of negative emotions. Nowadays college students are often plagued by academic pressure, interpersonal problems, and emotional confusion. How to effectively reduce negative emotions and reduce the incidence of psychological problems in “adversity” has aroused in-depth discussions among scholars. Allen pointed out that the response and encouragement given by parents to their kids and the ability of adolescents to acquire their parents’ affection for themselves in the family play a positive role in the development of their independence (63). In a positive parent-child relationship, parents usually take a guiding and caring attitude towards their children’s development, they are more concerned with communication with their children and building trust than making rules. Simultaneously, a harmonious family atmosphere, encouragement, and affirmation for children not only help to improve children’s growth skills, but also enhance their sense of self-identity. These will help children cope with difficulties with more confidence and calmness when facing the adversity in the future, help them learn from the experience of difficult situations to eventually achieve their personal growth. Negative parent-child relationships tend to be relatively distant the relationship between parents from children, with parents not paying enough attention to their children’s needs, leading children feel more alienated from their parents. In this state, individuals are prone to negative emotions in the face of adversity, lack self-confidence, and reduce opportunities for personal growth.

What we didn’t expect was that college students’ depression scores were neither correlated with parent-child attachment scores nor psychological resilience scores. Whereas previous studies had found that psychological resilience scores were negatively correlated with depressive symptoms (64–66). These previous studies showed that the improvement of psychology resilience can alleviate the depressive symptoms of college students, meanwhile, psychological resilience also mediates and regulates the relationship between other influences and depression.

It has also been noted that the level of parent-child attachment is positively correlated with the seriousness of depression. High levels of parental attachment may lead to higher levels of depression in college students at the end of the semester after entering college (67), secure parent-child attachment styles are associated with lower susceptibility to depression, and maternal alienation may lead to an increased susceptibility to depression (67), which is not in line with our findings. This discrepancy may due to previous studies usually take samples from specific populations, their samples are characteristics of small sample size and relatively single population, and lack universality.

The causes of depression are complex, involving biological, psychological and social factors. Although parent-child attachment and psychological resilience have a positive impact on mental health, they may not be the main drivers of depression. Depression may be more related to genetics, neurobiochemistry, environmental stressors (e.g., academic and employment pressure), and lack of social support. For example, a previous study found that parent-child attachment (especially estrangement from the mother) had a significant predictive effect on the dimensions of college students who are depression-susceptible personality, such as sensitivity, competitiveness, and closed-off defensiveness, but did not significantly affect the “rigorous and serious” dimension, indicating that the effect of parent-child attachment on depression has limitations (67). In addition, existing studies had pointed out that college students have gradually separated from their families, and the impact of social support networks (such as peer relationships) on mental health has gradually increased, and the direct impact of family attachment may be weakened (68). This also suggested that the effect of parent-child attachment on depression is limited. Based on these findings, we agree that the central mechanism of parent-child attachment on depression in college students is that parent-child attachment serves more as an emotional ‘safe base’, rather than directly moderating the causes of depression.

Psychological resilience helps individuals cope with stress, but depression may stem from deeper emotional or cognitive issues, such as low self-worth and negative thought patterns, which may be beyond the scope of regulation. A previous study suggested that psychological resilience only partially mediated the relationship between childhood trauma and depressive episodes, but that deeper trauma (e.g., lack of self-worth) may be beyond the moderating capacity of psychological resilience (69). A previous research (70) revealed that psychological resilience can alleviate anxiety through cognitive restructuring, but the cognitive characteristics of depressed individuals are characterized by negative attributional styles, and they develop stable negative thought patterns that are difficult to regulate through psychological resilience. These negative attributions include: internal attribution (‘it’s my fault’), stable attribution (‘it will never change’), and global attribution (‘it affects all parties’). Even if the intervention of psychological resilience attempt to introduce individuals’ cognitive restructuring, attribution bias in individuals with depression can still hinder its effectiveness (71). In addition, their “affective forecasting bias” make them underestimate the likelihood of a positive experience in the future, further solidifying the negative thinking. A previous study (72) revealed that the biological mechanism of psychological resilience relies on the functioning of specific brain regions (e.g., thalamo-cortical loop), but the chronic stress may lead to the failure of this loop, so that even highly resilient individuals may still fall into depression. Suppose that the core mechanism of psychological resilience on regulating depression in college students is primarily through dynamic coping with short-term stress, whereas depression often involves stable cognitive biases (e.g., self-denial) or neurobiochemical imbalances (e.g., abnormal serotonin levels) (69, 72). It is well known that major life events, such as long-term unemployment may break the ‘threshold’ of psychological resilience, leading to a breakdown of regulatory mechanisms and limit its modulation (70).

Anxiety is often associated with uncertainty about the future and stressful events (e.g., employment pressure, academic competition). The parent-child attachment can more directly alleviate such immediate stressors by providing a sense of security (e.g., emotional support) with coping resources (e.g., family support). Meanwhile, psychological resilience can reduce anxiety reactions through cognitive reframing (e.g., take stress as a challenge). However, depression is often associated with a lack of sense of self-worth, chronic emotional neglect, or trauma, which may be beyond the regulation of parent-child attachment and psychological resilience. Thus, parent-child attachment and psychological resilience may more directly alleviate anxiety and have a weaker effect on depression. This seems to suggest the etiology and pathogenesis of depression and anxiety among college students may be different. Future studies need to further explore other potential influencing factors on depression among college students, such as social support, interpersonal communication, excessive smartphone use, life events, and cultural background, etc., to understand the causes of depression among college students more comprehensively.

## 5 Strengths and limitations

Our study is based on samples of 100,253 college students in China, the sample size is larger, the reliability is relatively good, and the findings are relatively universal. However, we must acknowledge that our research has some limitations. Firstly, this study employed a cross-sectional and non-experimental design, thus it is unable to draw any causal or directional conclusions. In the future, longitudinal research or experimental methods should be adopted to more accurately reveal the changing directions of these interrelationships. Secondly, the assessment tools we used are all self-assessment tools, which are very common and have high reliability, a certain degree of caution is still needed. In the future, it might be possible to conduct researches by integrating multiple sources of reports and even objective indicators from laboratories, to enhance the accuracy of variable measurement. Thirdly, our attachment scale did not include peer-attachment. Previous research indicated that anxious attachment to a best friend was associated with more depressive symptoms (73). Future research on college students should consider incorporating peer attachment for a more in-depth study. Fifthly, our investigation did not include any information about peer support or interpersonal relationship. Previous study indicated that the peer support or good interpersonal relationships can substantially mitigate negative emotions, and are inversely correlated with depressive symptoms among university students (74–76). Future studies also necessary to conduct in-depth studies on effects of peer support or interpersonal relationships on the mental health of college students. Sixthly, previous study revealed that excessive smartphone use is strongly linked to depression and anxiety (77), while our survey did not include this profile. Future research should adopt validated tools to in-depth explore and analyze the psychological mechanisms underpinning the excessive smartphone use. Lastly, our results were based on a sample of Chinese college students, which is not yet clear whether they can be applied to the clinical population. It is necessary to conduct further research in the future to determine the generalization of our findings among different developmental stages, geographical regions, and cultural backgrounds.

## 6 Implications and conclusions

Our findings of the current study should not independently guide intervention ‘s strategy; however, when considering it within the context of the actual current situation regarding the mental health consequences of college students and within the broader framework of existing literature, its practical significance can be inferred. It is plausible that interventions promoting parent-child relationships, encouraging physical activity and exercise, developing a healthy lifestyle pattern, fostering positive attitude towards mental health and supporting individuals struggling with mental disorders could benefit youth with depression and anxiety symptoms. The collaborative efforts among parents, educators, medical professionals, and policymakers may be extremely necessary for addressing the complex issue of mental health and mitigating its detrimental effects on the physical and mental well-being of young people.

Our core findings highlight a significant negative correlation between parent-child relationships, psychological resilience and symptoms of anxiety, deepening our understanding of the psychological health challenges in the current era. Surprisingly, no such correlation was found in terms of depressive symptoms. Importantly, this research is the first time to conducted in a large sample, to clarify the relationship between parent-child relationships, psychological resilience and mental health, providing new perspectives for prevention and intervention.

Notably, the finding that no correlation between parent-child relationships, psychological resilience and depressive symptoms requires professionals to dig over the underlying mechanism of depressive symptoms. On the one hand, it is necessary to conduct in-depth research on the correlation between parent-child relationships, psychological resilience and depressive symptoms to further verify the findings of this study. On the other hand, it is also necessary to study the social and psychological factors that lead to depressive symptoms based on a broader perspective and a deeper scope.

In conclusion, this study not only provides new evidence regarding the correlation between parent-child relationships, psychological resilience and anxiety symptoms among college students, but also raises new questions for further in-depth research and rethinking of the mechanism underlying the occurrence of depression symptoms among college students. It highlights the importance of considering that different symptoms of individuals may require different intervention measures when implementing mental health interventions.

## Data Availability

The raw data supporting the conclusions of this article will be made available by the authors, without undue reservation.
